# Midazolam Efficacy Against Acute Hydrogen Sulfide-Induced Mortality and Neurotoxicity

**DOI:** 10.1007/s13181-017-0650-4

**Published:** 2018-01-09

**Authors:** Poojya Anantharam, Dong-Suk Kim, Elizabeth M. Whitley, Belinda Mahama, Paula Imerman, Piyush Padhi, Wilson K. Rumbeiha

**Affiliations:** 1 Department of Veterinary Diagnostic and Animal Production Medicine, Iowa State University, Ames, IA USA; 2 Pathogenesis, LLC, Gainesville, FL USA

**Keywords:** Hydrogen sulfide, Neurotoxicity, Neurodegeneration, Acute toxicity, Translational model

## Abstract

Hydrogen sulfide (H_2_S) is a colorless, highly neurotoxic gas. It is not only an occupational and environmental hazard but also of concern to the Department of Homeland Security for potential nefarious use. Acute high-dose H_2_S exposure causes death, while survivors may develop neurological sequelae. Currently, there is no suitable antidote for treatment of acute H_2_S-induced neurotoxicity. Midazolam (MDZ), an anti-convulsant drug recommended for treatment of nerve agent intoxications, could also be of value in treating acute H_2_S intoxication. In this study, we tested the hypothesis that MDZ is effective in preventing/treating acute H_2_S-induced neurotoxicity. This proof-of-concept study had two objectives: to determine whether MDZ prevents/reduces H_2_S-induced mortality and to test whether MDZ prevents H_2_S-induced neurological sequelae. MDZ (4 mg/kg) was administered IM in mice, 5 min pre-exposure to a high concentration of H_2_S at 1000 ppm or 12 min post-exposure to 1000 ppm H_2_S followed by 30 min of continuous exposure. A separate experiment tested whether MDZ pre-treatment prevented neurological sequelae. Endpoints monitored included assessment of clinical signs, mortality, behavioral changes, and brain histopathological changes. MDZ significantly reduced H_2_S-induced lethality, seizures, knockdown, and behavioral deficits (*p* < 0.01). MDZ also significantly prevented H_2_S-induced neurological sequelae, including weight loss, behavior deficits, neuroinflammation, and histopathologic lesions (*p* < 0.01). Overall, our findings show that MDZ is a promising drug for reducing H_2_S-induced acute mortality, neurotoxicity, and neurological sequelae.

## Introduction

Hydrogen sulfide (H_2_S) is an extremely toxic gas and is only second to carbon monoxide as a leading cause of gas-induced deaths. It is a hazard in many occupational settings where accidental acute high-dose exposure may occur following industrial malfunction or because of nefarious acts. Mass civilian casualties of acute H_2_S poisoning have occurred in the past [1, 2]. Because of its history as a chemical weapon before, there is concern about potential misuse of H_2_S in acts of terrorism, especially in confined spaces such as the massive underground railroad system or in high-rise buildings [3, 4]. At high concentrations, H_2_S rapidly exerts its toxic effects not only on the central nervous system but also on the respiratory and cardiovascular systems [5, 6]. Clinical signs of acute H_2_S poisoning include dyspnea, anxiety, restlessness, and ocular and upper respiratory tract irritations in moderate concentrations. Sudden collapse (“knockdown”) accompanied by unconsciousness, seizures, and breathing difficulty from pulmonary edema, arrhythmia, and hypotension are signs of acute exposure at higher concentrations.

Acute H_2_S poisoning causes high acute mortality, characterized by a steep concentration-response curve. At least 50% of H_2_S-induced deaths occur during exposure, while the remainder of the mortality of intoxicated victims occurs within 48 h of rescue [2]. A unique characteristic of this toxic gas is the “knockdown” associated with sudden exposure to high concentrations. This is an incapacitating effect, rendering the victims unable to escape [7]. Despite the high mortality, some victims of acute H_2_S poisoning survive with or without supportive treatment. However, some of the survivors of acute intoxication may develop long-term neurological sequelae characterized by psychiatric disturbances, persistent headaches, sleep disorders, anxiety, memory loss, learning disorders, hearing impairment, and movement disorders such as ataxia [5, 6, 8–10]. These and other neurological sequelae typically develop in victims who succumb to knockdown and coma for at least 5 min, but typically for 10–15 min. These neurological complications may or may not be permanent but can be incapacitating, leading to work disability. Currently, the exact mechanisms by which these neurological sequelae develop are not known.

Because most deaths occur at the scene, there is a critical need for a drug or drugs that can be used in the field for treatment of victims of acute H_2_S poisoning at the site. Currently, there is no Food and Drug Administration (FDA) approved drug for treatment of victim of acute H_2_S poisoning in the field. Currently recommended treatments of acute H_2_S poisoning are of questionable efficacy and cannot be effectively used in the field for treatment of mass casualties. For example, treatment recommendations include nitrite and hydroxocobalamin, both of which require intravenous (IV) injections [11–14]. Intravenous injections can be challenging to use in mass civilian victims in the field. Besides, IV nitrite injections are associated with hypotension, a limiting side effect [1]. Also, although hydroxocobalamin binds H_2_S, large volumes of IV hydroxocobalamin are recommended. Cobinamide (Cob) is a promising experimental H_2_S countermeasure that showed efficacy in animal models following intramuscular injection [14, 15]. However, Cob has not been approved by the FDA yet. Nitrite, hydroxocobalamin, and cobinamide all largely work by binding H_2_S in vivo. Given that H_2_S rapidly transmutes to the hydrosulfide ion, which in turn is rapidly metabolized to thiosulfate and sulfate, the therapeutic window for drugs that bind sulfide is very narrow [16, 17]. Consequently, there is a need to develop countermeasures with different mechanisms of action that can easily be used in the field for treatment of mass civilian casualties.

Midazolam (MDZ), a common benzodiazepine and an anti-seizure medication, is on the list of The World Health Organization most essential drugs [18]. It is available worldwide for treatment for epilepsy and seizures and has recently shown promise as a countermeasure against nerve agent-induced neurotoxicity [18]. MDZ is also a powerful anxiolytic and has sedative and amnestic properties. Due to its rapid onset (5–10 min), relatively short half-life, and efficacy for treatment of acute seizures and status epilepticus, MDZ is currently being considered to replace diazepam in the strategic defense stockpile as an anti-convulsant for nerve agent exposure [18]. It is very water-soluble and therefore readily absorbed by intramuscular (IM) injection [18]. Maximum plasma concentration is reached in about 30 min post-IM injection with > 90% bioavailability [18, 19]. MDZ has high affinity for the benzodiazepine receptor and its anti-seizure activity is believed to arise from its potentiation of synaptic GABA_A_ receptors [18]. Due to these desirable properties, we hypothesized that MDZ is effective for treatment of acute H_2_S-induced neurotoxicity by suppressing H_2_S-induced seizure effects. This followed our previous observations in the mouse model that deaths followed intense seizure activities [20]. This observation is similar to that of O’Donoghue in a pig study of acute H_2_S poisoning [21]. The objective of this proof-of-concept study was to conduct a series of experiments to test the hypothesis that MDZ is efficacious for treatment of acute H_2_S-induced mortality and neurotoxicity. This is a groundbreaking study because no prior studies have addressed this question.

## Material and Methods

### Animals

All animal studies were approved by the Iowa State University Institutional Animal Care and Use Committee (IACUC). The 7–8-week-old C57/BL6 male mice used in these studies were purchased from The Jackson Laboratories (Bar Harbor, ME) and weighed 20–25 g at the beginning of the experiment. Mice were housed five per cage in the Laboratory Animal Resource (LAR) Facility of the Iowa State University College of Veterinary Medicine (ISU CVM, Ames, IA). They were housed at a room temperature of 68–70 °F, relative humidity of 35–50%, and a 12-h light/dark cycle. They were provided 14% Protein Rodent maintenance diet (Teklad HSD Inc., WI, USA) and drinking water ad libitum. Mice were acclimated for 1 week prior to the start of the studies.

### Experimental Approach

In this proof-of-concept study, we conducted a series of experiments to evaluate the efficacy of MDZ for prophylactic treatment (pre-H_2_S exposure) and for treatment of acute H_2_S exposure (during exposure). Fully conscious and freely moving mice were utilized. The mice were exposed to H_2_S by whole body inhalation exposure, details of which have previously been published [20]. Briefly, the experiments were conducted under a chemical fume hood approved by the Environmental Health & Safety at the ISU. H_2_S was introduced to the chamber, and the desired concentration was achieved by dilution with normal breathing air from a gas cylinder. The concentration of H_2_S in the exposure chamber was constantly monitored using a H_2_S monitor (Environmental Equipment and Supply, Harrisburg, PA) that was custom designed to measure concentrations of up to 1000 ppm of H_2_S.

#### Objective 1: To Test the Efficacy of Midazolam for Reducing H_2_S-Induced Acute Mortality

##### Experiment 1

**Fig. 1:**
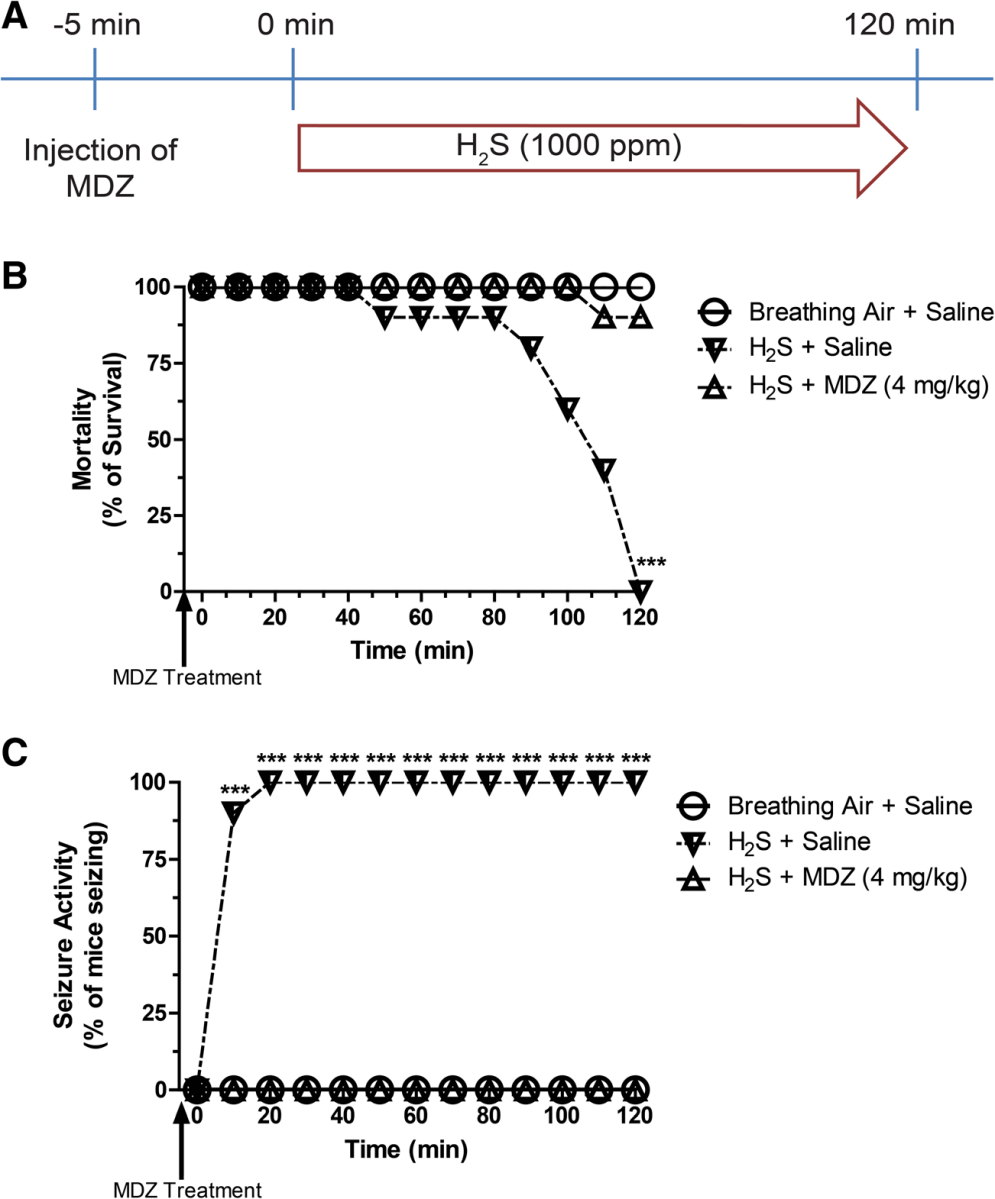
**a** Treatment paradigm to determine the prophylactic efficacy of MDZ. **b** MDZ prevented H_2_S-induced mortality by 90%. The survival data between H_2_S + saline and H_2_S + MDZ groups is significantly different (*p* < 0.05, log-rank test, *n* = 10). **c** MDZ prevented H_2_S-induced seizure activity in mice. The seizure data between H_2_S + saline and H_2_S + MDZ groups is significantly different (*p* < 0.05, log-rank test, *n* = 10). Asterisks (***p < 0.001) indicate statistically significant difference between H_2_S + saline and the H_2_S + MDZ groups

In this experiment, we tested the hypothesis that injecting MDZ prophylactically before a single high-dose H_2_S exposure reduced mortality. Mice were injected once, either with 0.9% saline or MDZ (4 mg/kg), IM 5 min prior exposure to 1000 ppm H_2_S for 120 min (Fig. 1a). This dosage is similar to that (0.5–10 mg/kg IM) used in experimental studies where MDZ was investigated for treatment of seizures induced by nerve agents in guinea pigs and rats [18].

##### Experiment 2

**Fig. 2:**
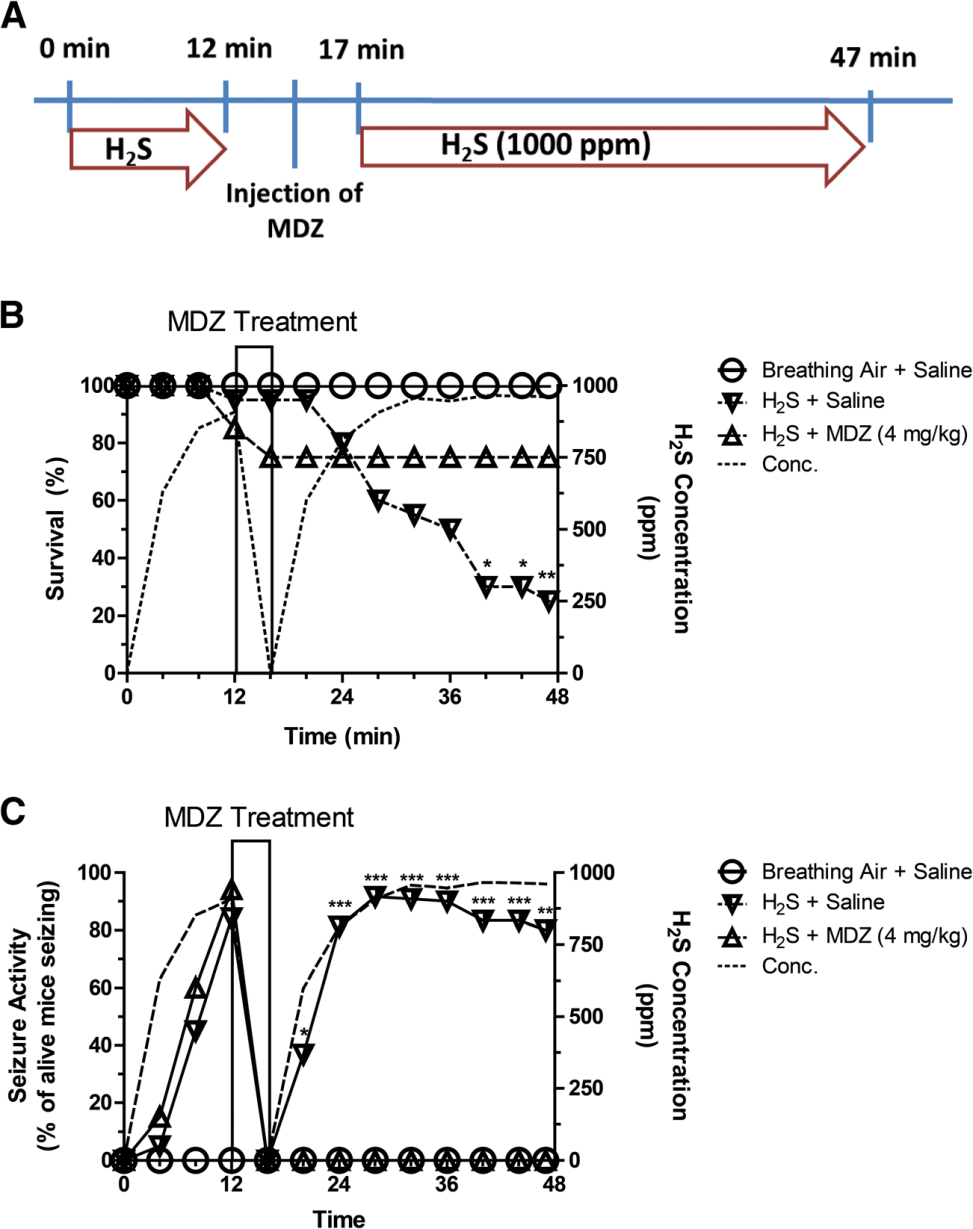
**a** Treatment paradigm to determine the efficacy of MDZ for treatment of H_2_S-induced neurotoxicity given during H_2_S exposure. **b** Following MDZ treatment, no more mice died compared to about 20% survival for saline-treated mice. The survival curve between H_2_S + saline and H_2_S + MDZ groups is significantly different (*p* < 0.0001, log-rank test, *n* = 20). **c** MDZ abolished H_2_S-induced seizure activity in mice (*n* = 20). Asterisks (*p < 0.05, **p < 0.01, ***p < 0.001) indicate statistically significant difference between H_2_S + saline and the H_2_S + MDZ groups

In this experiment, we tested the hypothesis that MDZ given once during acute high-dose H_2_S exposure reduces H_2_S-induced mortality. Mice were exposed to 1000 ppm H_2_S for 12 min in the inhalation chamber, after which mice were removed for injection of MDZ (4 mg/kg bw) or saline (0.9%) IM. All IM injections were 50 μL in the gastrocnemius muscle. Immediately after MDZ or saline injection, mice were returned to the inhalation chamber for continued exposure to H_2_S (1000 ppm) for 30 min. Mice were constantly observed during exposure for clinical signs of intoxication using a modified functional observation battery (FOB) [20, 22]. Specifically, seizure, knockdown, and time of death were noted (Fig. 2a). This exposure paradigm was done to simulate rescue from underground confined spaces or from high-rise buildings where victim will be treated upon arrival of first responders, which was estimated to take about 10 min, but complete evacuation may last another half an hour. The difference is that in our model, we removed the mice from the chamber to inject them because our exposure chamber is not designed to allow safe injections to be done while the mice are in the chamber. Mice were immediately returned to the chamber and H_2_S exposure immediately resumed. This procedure was completed within 5 min.

#### Objective 2: To Test the Efficacy of Midazolam for Preventing Neurological Sequelae

**Fig. 3:**
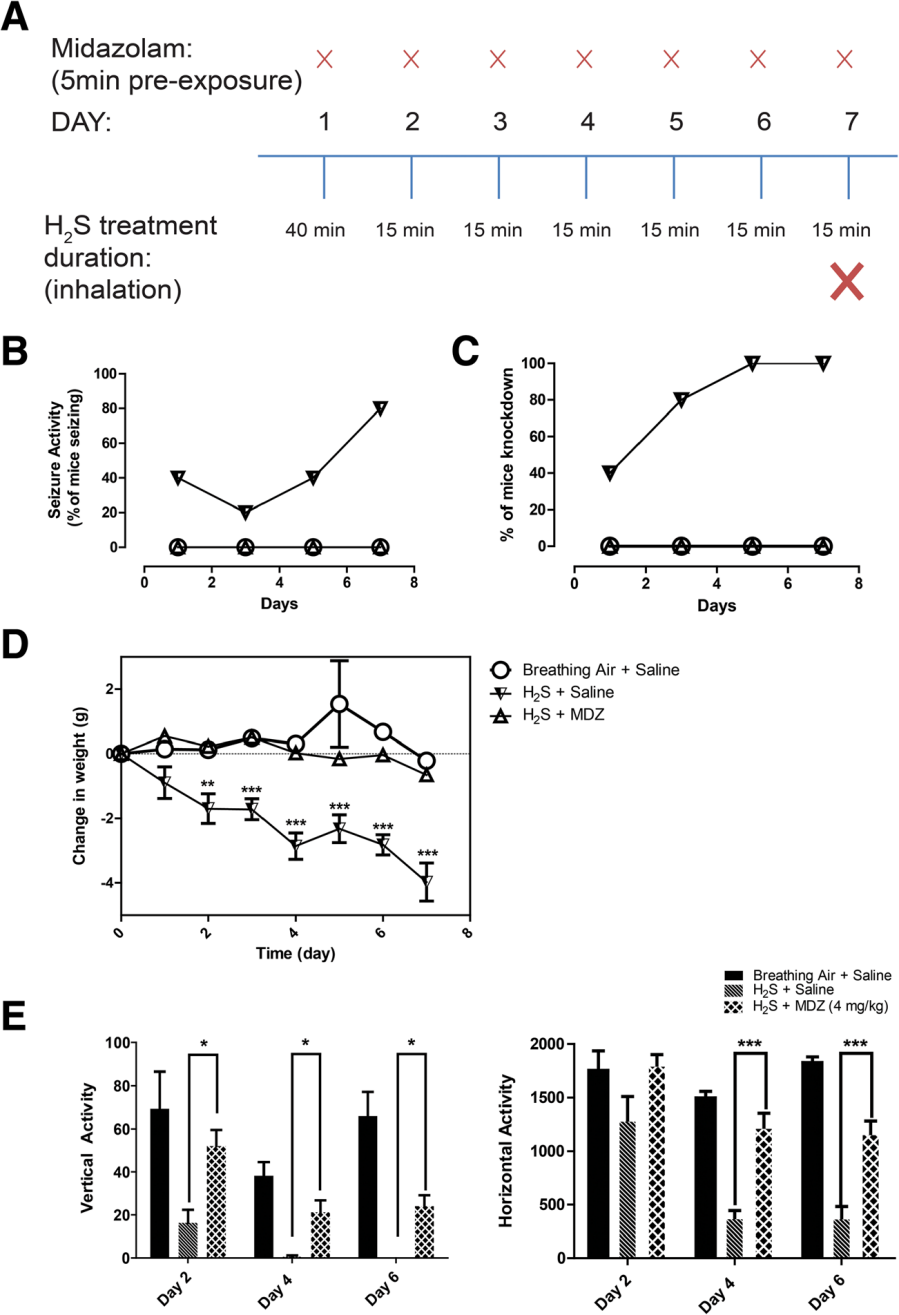
**a** Summary treatment paradigm of H_2_S-induced neurological sequelae in mice prophylactically treated with MDZ. **b** MDZ completely prevented seizure activity and knockdown (**c**) consistently during the entire exposure period (*n* = 5). Seizure and knockdown were presented as percentage to breathing air control group. Seizure and knockdown data were not statistically analyzed due to the possibility of multiple seizure and knockdown from same mice during repeated exposure to H_2_S. **d** Mice exposed to H_2_S and injected with saline lost statistically significant more weight compared to the breathing air controls injected with saline. MDZ prophylactically prevented H_2_S-induced weight loss (*n* = 5). **e** MDZ prevented H_2_S-induced motor deficits (*n* = 5). Graphs are represented as mean values. *p < 0.05, **p < 0.001, ***p < 0.001, two-way ANOVA followed by Bonferroni’s post-test between H_2_S + saline and H_2_S + MDZ groups

In this proof-of-concept experiment, we used a MDZ/H_2_S exposure paradigm summarized in Fig. 3a. Briefly, we tested the hypothesis that MDZ administered prophylactically 5 min prior to H_2_S exposure prevents H_2_S-induced neurological sequelae. The justification for repeated short-term exposures has been provided in prior publications [20]. Briefly, some of the human survivors of single acute high-dose H_2_S poisoning develop neurodegeneration and other neurological sequelae. Whereas a typical exposure scenario in humans is to one large H_2_S exposure leading to neurodegeneration, this approach is characterized by very high acute mortality in mice during exposure, with only a few of the surviving mice developing neurodegeneration [23]. Using the single-exposure approach, as occurs in humans, requires an unreasonably large number of mice to test the hypothesis to achieve a statistically satisfactory level of significance. We found that repeated short-term acute exposures to H_2_S to be a more humane approach because it is associated with lower mortality than one-time exposure paradigm and yet yields brain lesions recapitulating the human condition [20]. Currently, there is no other animal model which recapitulates the H_2_S-induced neurodegeneration following a single acute exposure by inhalation. A repeat short-term exposure approach was also used in a monkey study by Lund and Wieland [24]. In their study, monkeys exposed to high doses died and only those given short-term repeated exposures manifested lesions reminiscent of the human condition. This is the same approach we took in this and previous studies to induce neurodegeneration in this mouse model of H_2_S-induced neurodegeneration [20, 23, 25].

Mice were divided into three different groups of five male mice as follows: Group 1 mice were injected with 0.9% saline 5 min before exposure to normal breathing air from a cylinder; mice in Group 2 were injected with 0.9% saline 5 min prior to exposure to 765 ppm H_2_S; Group 3 mice were injected with MDZ (4 mg/kg bw) 5 min prior to exposure to 765 ppm H_2_S. MDZ or 0.9% saline was injected in the rear leg (gastrocnemius) muscle in 50 μL of solution. Normal breathing air and H_2_S were delivered from gas cylinders. In this acute repeated H_2_S exposure paradigm, on day 0, mice were exposed to 765 ppm H_2_S or breathing air for 40 min post-injection of saline or MDZ as described above. On subsequent days, the same groups of mice were exposed either to 765 ppm H_2_S or to normal breathing air for 15 min only post-injection with 0.9% normal saline, each day for 6 days.

#### Objective 3: To Test the Effect of H_2_S on Brain Midazolam Concentrations

During preliminary studies, we observed clinical differences between mice injected MDZ with or without exposure H_2_S. Specifically, given equivalent dosages of MDZ, the sleeping time of mice exposed to H_2_S was longer than that of mice without exposure to H_2_S. We hypothesized that high-dose acute H_2_S exposure causes higher MDZ levels in brains mice exposed to H_2_S. In order to test this hypothesis, two groups of mice were exposed to 1000 ppm H_2_S for 20 min. They were then removed from the inhalation for chamber for 5 min during which mice were injected with 4 mg/kg bw midazolam. Mice were then placed back in the inhalation chamber for another 95 min (Fig. 6a). A breathing air group of mice injected with saline was used as a negative control. Upon termination of H_2_S/breathing air exposure, mice were removed from the chamber, immediately decapitated, and their brains were rapidly removed and placed on ice. After necropsy, brain tissues were subsequently stored at − 80 °C until ready for analysis. For this proof-of-concept experiment, only brain tissue was analyzed.

### Clinical Assessment

To obtain baseline data, animals were evaluated clinically and weighed starting 3 days prior to H_2_S exposure. Mice were weighed daily until euthanasia. In addition, a modified FOB was used to evaluate clinical signs during H_2_S exposure, including knockdown, seizure activity, abnormal gait, and autonomic function, such as urination and defecation. The same trained observer, who conducted the study, assessed the mice throughout the entirety of the experiment.

### Behavioral Testing

For behavioral assessment, we used the VersaMax open-field test. Behavior assessments for open-field activity were performed 3 h after mice were exposed to H_2_S. This was performed on days 2, 4, or 6 as previously described [20]. Briefly, an automated computer-controlled device (Model RXYZCM-16; Accuscan, Columbus, OH, USA) was used to measure the spontaneous activity of mice in this open-field test. The dimensions of the activity chamber were 40 × 40 × 30.5 cm, made of clear Plexiglas and covered with a Plexiglas lid with holes for ventilation. Data was collected and analyzed by a VersaMax Analyzer (Model CDA-8; AccuScan). Mice were acclimated to the chamber 2 days before H_2_S exposure. On test days, mice were placed inside the infrared monitor for 2 min to acclimate to the chamber. Open-field activities were recorded for 10-min test sessions assessing multiple parameters, including vertical activity and horizontal activity.

### Histopathology and Immunohistochemistry

Mice designated for histopathology were euthanized 24 h after the last H_2_S exposure using a previously published procedure that employed a cocktail of 100 mg/kg bw ketamine and 10 mg/kg bw xylazine given intraperitoneally [20]. Briefly, once the mice were in a surgical plane of anesthesia, the thorax was opened and fresh 4% paraformaldehyde solution (PFA, pH 7.4) was injected through the left ventricle to perfuse the animal. Thereafter, brains were post-fixed in 4% PFA for 24 h, processed routinely, paraffin embedded, sectioned at 5 μm, and stained with hematoxylin and eosin for routine histopathology. Additional brain sections were stained using an indirect immunostaining protocol (Vectastain Elite ABC kit, PK-6101, Vector Laboratories, Inc., Burlingame, CA) that employed primary antibodies directed against glial fibrillary acidic protein (GFAP, ab72600, Abcam) or inducible nitric oxide synthase (iNOS, ab15323, Abcam). Diaminobenzidine (DAB, SK-4100, Vector Laboratories, Inc.) was used for a chromogen. Stained sections were examined microscopically using a Nikon Eclipse Ci-L microscope with DS-Fi2 camera. Routine histopathology was conducted by a board-certified veterinary pathologist blinded to the study design. The semi-quantitative scale used for scoring the severity of lesions has been previously published [5].

### Analysis of Brain Midazolam

Whole brain tissue samples were individually minced uniformly with scissors. A 0.1 g brain tissue sample was weighed for extraction. A matrix standard curve was also prepared using 4–0.1 g control brain tissue samples containing 0, 0.1, 1, and 10 ng MDZ. MDZ was extracted according to Bjorkman et al. by adding 0.4 ml of 0.01 N hydrochloric acid (HCl) to each sample. Each sample was then vortexed for 10 s and sonicated for 5 min. A 100 μL of 0.5 N NaOH was subsequently added to each sample and then vortexed for 10 s. Samples were further extracted with 0.5 ml ethyl acetate and vortexed for another 10 s. Samples were then centrifuged at 20,000 ×*g* for 5 min. The top layer of ethyl acetate was removed and placed into clean glass vials [26]. The ethyl acetate extraction was performed twice, and the extracts combined. The combined extracts were then dried down under nitrogen, re-solvated in 200 μL methanol, and vortexed for 10 s, before being quantified by LC-MS/MS, by injection of 20 μL out of the 200 μL extract. This analysis was performed on a Varian 310 LCMS triple quadrupole instrument using a positive ESI with a needle voltage of (+) 3500, a shield voltage of (+) 600, drying gas temperature of 325 °C, nebulizer gas at 50 psi, and drying gas at 30 psi. Detection ion used was 326–290.9 with a capillary voltage of 132 and collision energy of 21.5 V. Confirmatory ion used was 326–244 with a capillary voltage of 132 and collision energy of 20 V. Separation was performed on two Varian Prostar pumps equipped with a Varian 410 autosampler using a Polaris 5 μm C-18A column (150 × 2.0 mm) at a flow rate of 0.25 mL/min. The mobile phase contained 60% 10 mM ammonium acetate and 0.1% formic acid in methanol and 40% 0.1% formic acid. Retention time of the MDZ was 3.5 min [27, 28]. All samples were quantified against the matrix standard curve.

### Data Analyses

Data are presented as mean and standard error of the mean. Clinical toxicity during exposures was analyzed using linear regression. Survival and seizure curve data were analyzed using log-rank test. Fisher’s Exact Test for Count Data was used for proportional count analysis for each time point between H_2_S + Saline and H_2_S + MDZ groups. Body weight change and behavioral test data were analyzed using two-way ANOVA followed by a Bonferroni’s post-test. MDZ concentration data were analyzed using one-way ANOVA. Histopathology scores were analyzed using a Student’s *t* test comparing the H_2_S and saline-treated mice to the H_2_S and midazolam-treated mice. ANOVA tests and log-rank test were performed on Prism version 6 (GraphPad Prism Software, La Jolla, CA). Fisher’s Exact Tests for Count Data were performed using R software version 3. 3. 2 (https://www.r-project.org/). Data was considered statistically significant when the *p* value is lower than 0.05.

## Results

### Objective 1: Midazolam Prevented H_2_S-Induced Mortality

#### Experiment 1

This experiment evaluated the efficacy of MDZ given pre-exposure to H_2_S. In this study, 100% of mice injected with saline and exposed to H_2_S experienced seizures and died (Fig. 1b–c). In contrast, in the group of mice pretreated with midazolam, only 10% mortality was observed at the 2 h time point when the experiment was terminated, with none of these mice experiencing seizures (Fig. 1b–c).

#### Experiment 2

This study evaluated the efficacy of MDZ given during exposure to a single acute high dose of H_2_S. All of the mice exposed to breathing air and injected with 0.9% normal saline survived. Compared to this group, only 25% of mice exposed to H_2_S and injected 0.9% normal saline survived (Fig. 2b). H_2_S-induced mortality was time- and concentration-dependent. However, in the group of mice exposed to H_2_S and treated with MDZ, the survival rate was 100%, indicating treatment with MDZ significant prevented mortality from H_2_S-induced toxicity (Fig. 2a). Furthermore, none of the H_2_S-exposed mice treated with MDZ manifested seizure activity compared to 90% in the H_2_S/saline group (Fig. 2c).

### Objective 2: Midazolam Prevented H_2_S-Induced Neurodegeneration and Neurotoxicity

Control mice exposed to breathing air and treated with saline were completely normal for the entire duration of the study. During H_2_S exposure, mice pretreated with MDZ and exposed to H_2_S were clinically healthy compared to mice treated with saline. Specifically, saline pre-treated mice and exposed to H_2_S exhibited lacrimation, salivation, ataxia, impaired righting reflex (knockdown), and convulsions which were absent in mice pre-treated with MDZ. We considered mice in lateral recumbence with inability to right self as experiencing knockdown. Mice in knockdown could separately and distinctly manifest seizure activities on and off with continued H_2_S exposure. None of the mice pre-treated with MDZ manifested any seizures or knockdowns (Fig. 3b–c). However, MDZ-treated mice were less active and preferred to remain sedentary. MDZ also significantly prevented H_2_S-induced weight loss (Fig. 3d). The weights of mice from H_2_S/MDZ group were statistically similar to those of the saline/H_2_S group.

In the open-field test, mice pre-treated with MDZ performed statistically significantly better overall than mice in the saline/H_2_S group on all days of testing. The vertical and horizontal activities of mice pre-treated with MDZ were better by 55% or greater compared to the H_2_S and saline group (Fig. 3e).

**Fig. 4:**
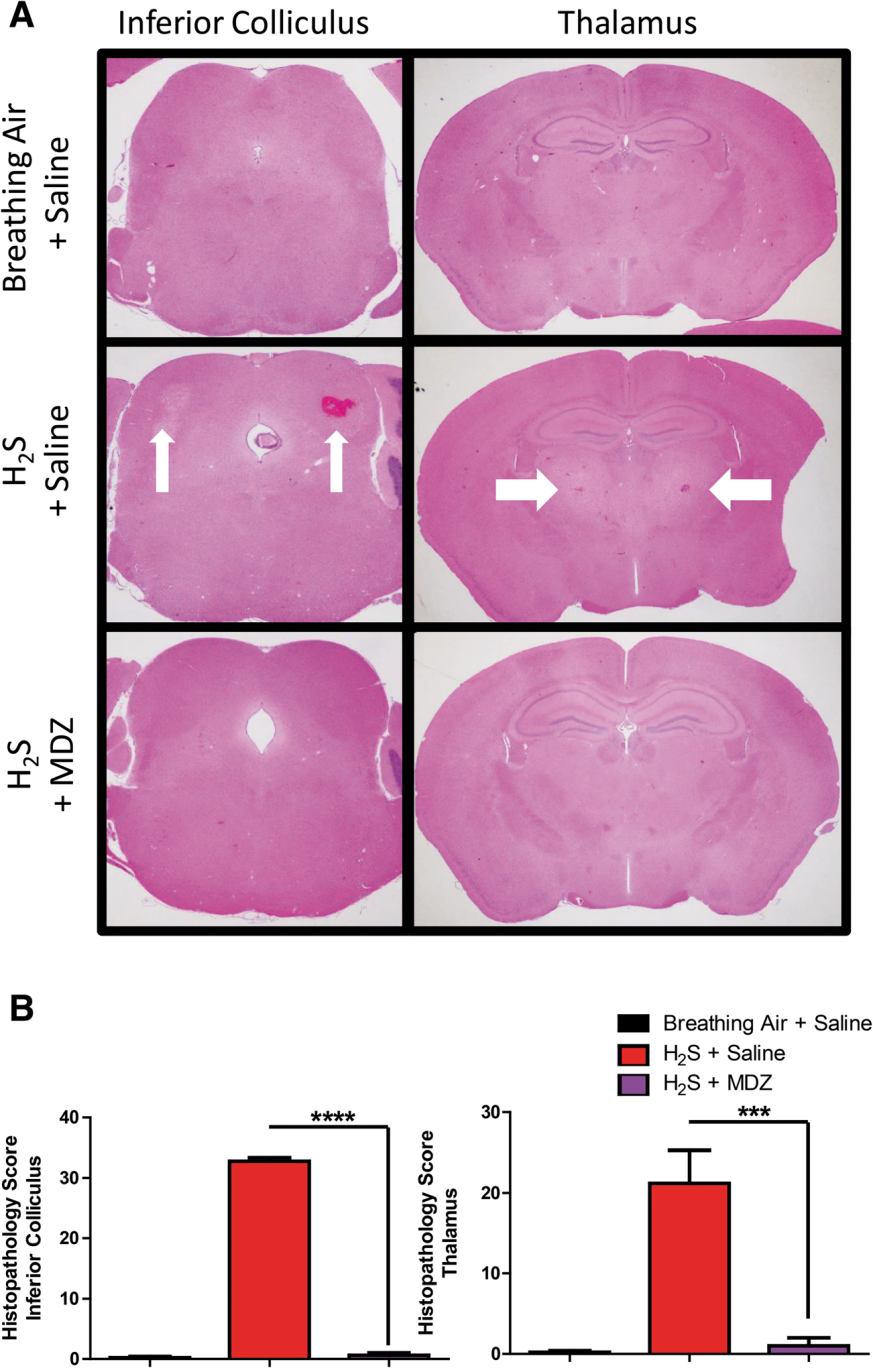
Photomicrographs of lesions in the thalamus and inferior colliculus of mice prophylactically treated with MDZ prior to H_2_S. Note the pallor and loss of neurons in the thalamus of the saline/H_2_S-exposed mouse. Note that brain tissue of the H_2_S + MDZ group is similar to that of the breathing air + saline group. Severe changes in the inferior colliculus of the H_2_S + saline-exposed mice include marked vacuolization of the neuropil, degeneration and loss of neurons, and prominent glial response. Graphs are represented as mean values. Asterisks (****p < 0.0001, ***p < 0.001) indicate a significant difference between H_2_S + saline and the H_2_S + MDZ groups

**Fig. 5:**
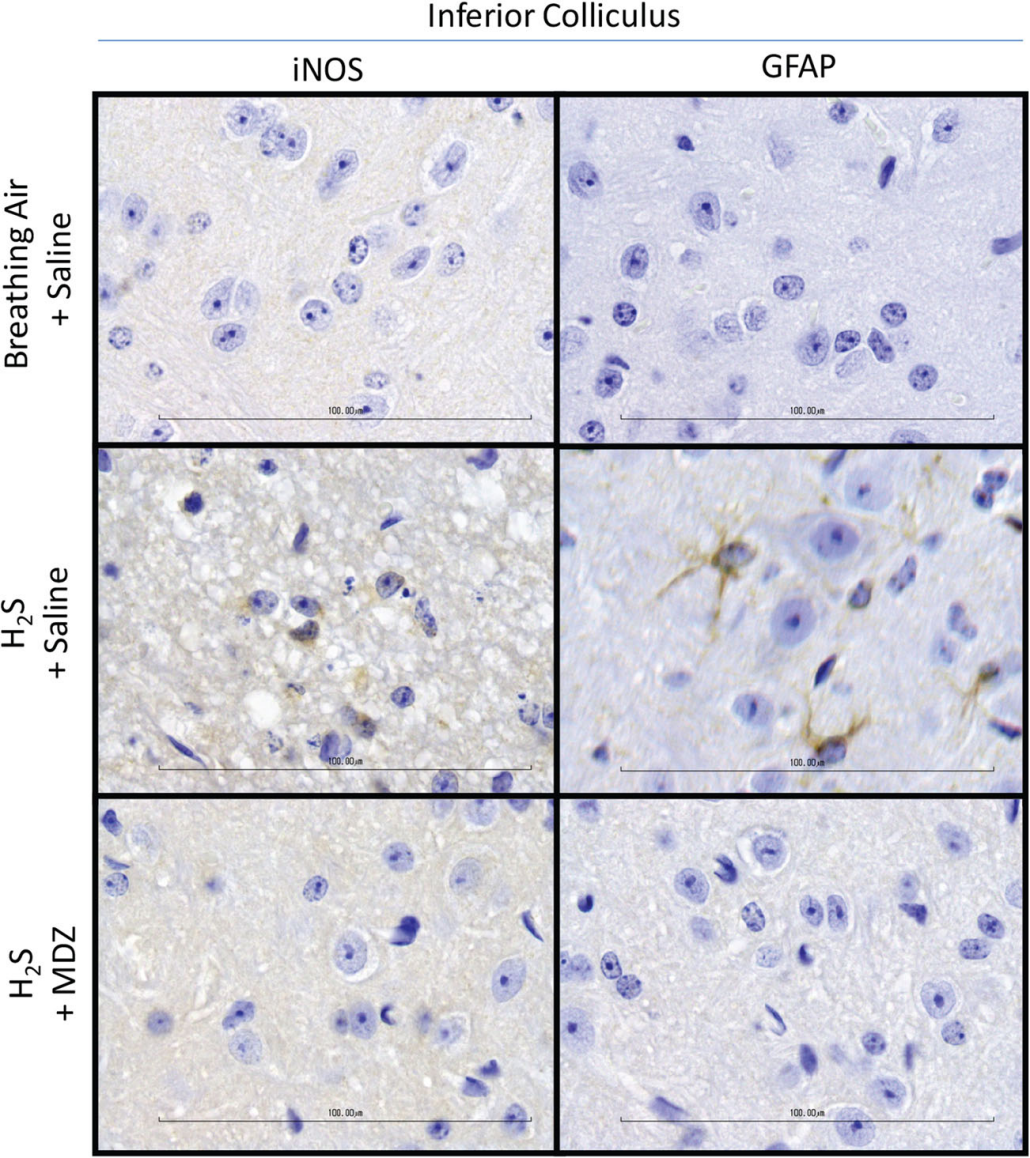
Representative photomicrographs of immunohistochemical staining of the inferior colliculus demonstrating expression of glial fibrillary acidic protein (GFAP), a marker of astrocyte activation, and inducible nitric oxide synthase (iNOS), a marker for neuroinflammation. Note the increased expression of GFAP and iNOS (brown chromogen deposition) in the brain of the saline/H_2_S group, while levels of these markers in the brains of MDZ-treated animals have less immunostaining, suggesting less inflammation the MDZ-treated group

Without MDZ pre-treatment, exposure to H_2_S consistently induced severe necrotic lesions in the inferior colliculus and thalamus, often with mild or moderate hemorrhage (Fig. 4a,b). Microscopically, the inferior colliculus of H_2_S-exposed mice showed extensive vacuolization of the neuropil, degeneration or loss of neurons, scattered apoptotic cell debris, influx and activation of microglia and astrocytes, and foci of hemorrhage in some animals. Pre-treatment with MDZ markedly reduced the incidence and severity of these neurologic lesions. The most consistent changes observed in the inferior colliculus or thalami of MDZ-treated mice were minimal to mild enlargement and prominence of microglial nuclei and vacuolization of the neuropil. Lesions were not observed in animals exposed to breathing air. Subjective assessment of intensity and distribution of immunopositivity in GFAP- and iNOS-immunostained sections revealed moderately increased expression of GFAP and iNOS in untreated, H_2_S-exposed mice and minimal to mildly increased GFAP and iNOS in MDZ-treated animals (Fig. 5).

### Objective 3: H_2_S Affects Brain Midazolam Concentration

**Fig. 6:**
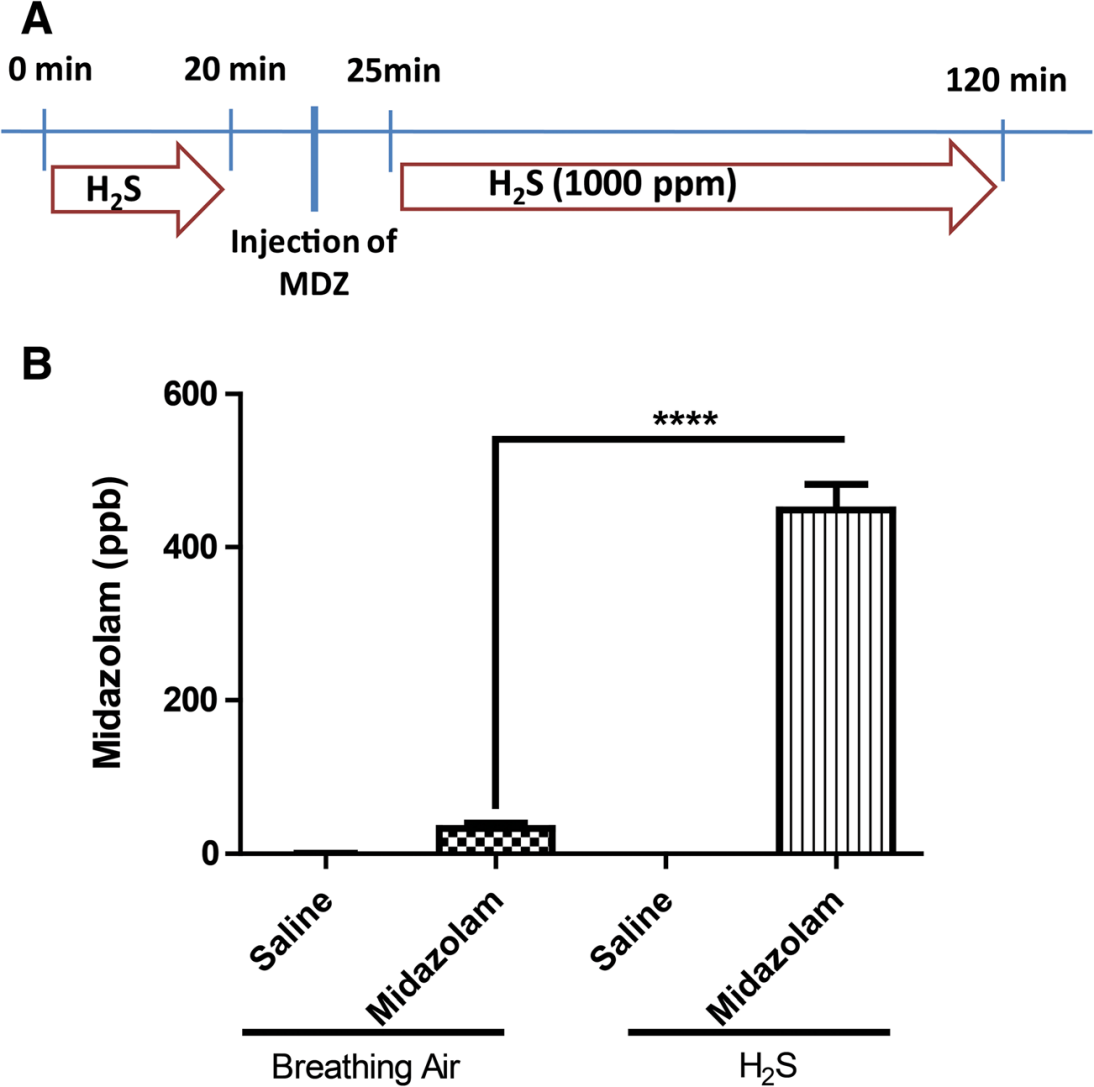
MDZ concentration in the brain. Note the significantly higher MDZ concentration in mice exposed to high concentration of H_2_S compared to those without H_2_S exposure. Graphs are represented as mean values. ****p < 0.0001, ANOVA followed by Bonferroni’s post-test between H_2_S + MDZ and the breathing air + MDZ group

This experiment evaluated the effect of H_2_S on MDZ brain concentration. We measured MDZ in brain tissue and found that mice exposed to breathing air and injected with MDZ had significantly less MDZ concentration in the brain than mice which were injected with MDZ and exposed to H_2_S (Fig. 6).

## Discussion

H_2_S is a rapidly acting, highly neurotoxic gas, with high acute mortality, usually at the scene of exposure. Currently, there is a need for drugs for treatment of victims of acute H_2_S intoxication in the field [7, 29]. This seminal proof-of-concept study has shown that prophylactic treatment with MDZ before H_2_S exposure and treatment with MDZ during H_2_S exposure significantly increases survival in mice exposed to lethal concentrations of H_2_S. The study also shows that prophylactic treatment with MDZ prevents H_2_S-induced neurodegeneration and neurological sequelae. These preliminary findings are significant considering that there is no FDA-approved drug with such properties for treatment of H_2_S poisoning now currently on the market.

The exact mechanism(s) by which MDZ was able to increase survival and to reduce neurodegeneration is/are not known and is beyond the objectives of this concept study. It is likely counteracting one or more of the effects of H_2_S-induced neurotoxicity. Inhibition of Complex IV in cytochrome c oxidase resulting in reduced ATP is a well-established mechanism of H_2_S-induced toxicity [15]. H_2_S also causes oxidative stress via generation of reactive oxygen and sulfur-free radicals [30, 31]. H_2_S also causes neurotoxicity by increasing concentrations of biogenic amines [20]. In this mouse model of H_2_S-induced neurotoxicity, we have previously reported that lethality was associated with increased seizure activity [20]. Mortality was also previously associated with seizures in a pig study [21]. Consequently, we hypothesized that suppression of seizures by MDZ increases survival in H_2_S-intoxicated mice. Results of this proof-of-principle study indicate that our hypothesis is correct. However, identifying which of the above neurotoxic mechanisms of H_2_S are antagonized by MDZ is beyond the objectives of this study. We hypothesize that, MDZ, an anti-convulsant drug, likely works by quieting neuronal activity through GABA_A_ receptors. MDZ potentiates GABA_A_ receptors, inhibiting excitability [18]. However, MDZ has also been shown to counteract oxidative stress [32, 33]. Specific mechanisms involved will be evaluated in future studies. MDZ is appealing because it can be given easily in the field for treatment of mass civilian victims of acute H_2_S poisoning IM by an autoinjector similar to an EpiPen®.

Until now, other therapeutics being evaluated or recommended for treatment of acute H_2_S poisoning, including nitrite and hydroxocobalamin, work by binding H_2_S in vivo*.* Treatments that bind sulfides have a disadvantage because H_2_S rapidly dissociates into daughter sulfide species almost instantaneously in vivo. For example, at the normal pH of 7.4, H_2_S dissociates 2:1 into hydrosulfide anion:undissociated H_2_S which exist in a dynamic at this ratio [15]. Furthermore, H_2_S is rapidly metabolized in the liver and kidney to thiosulfate and sulfate. It has been reported that 70% of H_2_S is metabolized to sulfate within 15 min [17]. Optimal efficacy of such drugs occurs when H_2_S is still available to scavengers. It is not surprising, therefore, that the efficacy of nitrite for treatment of sulfide toxicity is questioned [5]. Besides, both nitrite and hydroxocobalamin have to be given IV, a route not convenient for treatment of mass civilian casualties. MDZ, which is well-absorbed by IM route, acts rapidly [18, 19]. For example, in models of nerve agent intoxications, peak efficacy has been reported to occur within 10 min of IM injection [18]. MDZ also has the added advantage that it is currently approved as an anti-convulsant drug and is currently being considered for inclusion in the strategic defense stockpile for treatment of chemical-induced seizures, including nerve agents. Given the promising preliminary results, repurposing MDZ for acute treatment of acute H_2_S poisoning is attractive. Considering it is already approved for human use, should it prove safe for treatment of acute H_2_S intoxication, it will likely be brought to market much faster.

This preliminary data is encouraging because MDZ has significant potential for field application. For example, prophylactic treatment with MDZ could be an option for first responders before attempted rescue, as an added layer of security. Currently, first responders use self-contained breathing apparatus in rescue missions to avoid intoxication. Sometimes, these get dislodged and first responders get exposed to H_2_S [34, 35]. Given in appropriate doses which do not impair judgment or cognitive abilities in first responders, MDZ could potentially serve as an added layer of protection. However, such limitations are of less concern for treatment of civilian victims of acute H_2_S poisoning during or after H_2_S exposure.

The rapid absorption of MDZ following IM injection is particularly appealing, especially for field treatment of mass civilian casualties during accidents or terrorist acts. Persistent convulsions are one of the sequelae reported in severely affected victims of acute H_2_S poisoning. MDZ may potentially be useful for post-H_2_S exposure treatment in such patients. The ability of MDZ to prevent mortality when given before or during H_2_S exposure, as shown in this study, is phenomenal. Besides increasing survival, MDZ also significantly reduced H_2_S-induced neurodegeneration and resulted in improved behavioral performance. We also found that MDZ pre-treatment consistently prevented knockdown and seizures induced by high-dose acute exposures to H_2_S. The fact that MDZ pretreatment prophylactically prevented loss in body weight also suggests that these mice were clinically better than saline-treated control mice. It will be interesting in future studies to investigate whether post-H_2_S treatment with MDZ also affords protection and increases survival and/or reduces neurodegeneration and improves behavioral performance.

Histologic lesions observed in the brains of untreated animals exposed to H_2_S are consistent with those observed in our previous studies using a mouse inhalation model of H_2_S exposure that generates severe lesions and are similar to those reported in human patients [20]. Pre-treatment with MDZ reduced the development and severity of histologic lesions, reinforcing the clinical and behavioral observations in these mice. Reduced induction of GFAP and iNOS, markers of astrocyte activation and inflammation, respectively, in animals that were prophylactically pre-treated with MDZ supports the notion that MDZ prevents the induction of an astroglial response and activation of inflammatory pathways. We have previously shown that inflammation plays a role in H_2_S-induced neurotoxicity [20]. The mechanism(s) of action by which prophylactic treatment with MDZ reduced mortality and neurodegeneration is/are not known and cannot be ascertained from this limited proof-of concept study. However, it has been reported that MDZ reduces seizure activity by binding to the GABA_A_ receptors leading to allosteric potentiation of GABA-gated hyperpolarization of the cell, inhibiting excitability [18]. Although not determined for H_2_S, seizure activity has been linked to neurodegeneration following nerve agent exposure [36]. Reduced seizure activity is potentially one of the mechanisms by which MDZ was neuroprotective in this study.

MDZ has also been used for treatment of critically ill patients suffering from pathologic effects of oxidative stress, such as infection, hemodynamic instability, and hypoxia [33]. H_2_S-induced neurotoxicity is characterized by hemodynamic instability (hypotension) and hypoxia [37, 38]. H_2_S-induced neurotoxicity is also characterized by oxidative stress [5, 15]. There is evidence supporting the inverse correlation between MDZ and reactive oxygen species [33]. MDZ has been shown to interfere with the synthesis and release of nitric oxide and tumor necrosis factor-alpha [33]. MDZ also exerts protective effects during oxidative stress through the activation of Protein Kinase B (Akt) via phosphorylation in neuronal cells. Akt phosphorylation plays an important role in cell proliferation and cell survival [32, 33]. Potentially, these are some of the mechanisms worthy of investigating in future experiments designed to define neuroprotective mechanisms of acute H_2_S poisoning in this animal model.

Another interesting finding from this study is the potential interaction between H_2_S and MDZ. Exposure to lethal concentration of H_2_S increased brain MDZ concentration. The reasons(s) for this finding are not clear and cannot be determined from this study; but either H_2_S increased penetration of MDZ in the brain or it impaired MDZ metabolism in the brain. Whatever the reason, this finding has practical implications. Dose-response MDZ studies are needed to identify an ideal therapeutic dose. In this study, we chose to use 4 mg/kg bw based on results of preliminary studies and because this mouse dosage is almost equivalent to the human dosage of 0.33 mg/kg bw corrected for surface area [39]. The recommended dose in adults is two 10 mg ChemPack MDZ auto-injectors, which for a 60 kg person is equivalent to 0.33 mg/kg.

This initial proof-of-concept study has some limitations. A lot more work lies ahead before MDZ can be recommended for treatment of human victims of acute H_2_S poisoning.

Among the limitations, this was an exploratory study, and data was collected using a small number of animals and only using one species and sex—male mice. It will be helpful to repeat this study with a large number of mice of both sexes. It will also be necessary to repeat the study in a non-rodent species because species differences between humans and experimental animals exist. Showing efficacy in more than one species will increase confidence in results reported here. Also, although results of prophylactic pretreatment with MZD have relevance for first responders, a major need is to rigorously evaluate the efficacy of MDZ for treatment of civilian victims of H_2_S poisoning in the field. To this end, preliminary results showing increased survival, reduced seizure activity, and reduced knockdown in mice injected with MDZ during H_2_S exposure are very encouraging. This is particularly so because H_2_S-induced acute toxicity is uniquely characterized by a steep dose-response curve with high mortality during or soon after exposure as a major outcome. More research is needed to conclusively determine the efficacy of MDZ given during exposure and to evaluate its efficacy given post-H_2_S exposure, because this is what is most relevant for civilian use. Another limitation is that this study involved only one MDZ dosage. Appropriate dose-response MDZ studies need to be done to choose a dosage that is not only efficacious but also safe and with minimal side effects. MDZ is rapidly and well-absorbed trans-nasally and via the sublingual routes [40, 41]. Future studies will test the efficacy of MDZ given via these routes. The advantage of these routes is that they bypass the liver and are potentially “dose-saving” compared to the IM route and likely will be associated with fewer side effects, if any. Both sublingual and trans-nasal routes are also very attractive for field treatment of civilian casualties as they are easily accessible.

In summary, in this mouse model, MDZ treatment reduced mortality, seizure activity, and behavioral deficits and was neuroprotective against H_2_S-induced neurotoxicity. Results of this proof-of-concept study also revealed potential interaction between acute H_2_S exposure and MDZ because brain MDZ concentrations were significantly higher in H_2_S-exposed mice than those that were not. We acknowledge the limitations of this single study. However, results of this study strongly suggest that MDZ is a promising novel drug candidate for treatment of acute H_2_S-induced neurotoxicity and neurodegeneration. Noted benefits of MDZ of reduced acute mortality, reduced seizures, and knockdown given during H_2_S exposure are very appealing, and further research is recommended to test the efficacy of MDZ for treatment of acute H_2_S intoxication and for understanding its mechanisms of action against H_2_S-induced neurotoxicity.
